# *Mesomycoplasma hyopneumoniae* lipoprotein Mhp390 serves as a plasminogen receptor mediating extracellular matrix degradation and respiratory epithelial cells injury

**DOI:** 10.1186/s13567-025-01551-7

**Published:** 2025-06-21

**Authors:** Wei Liu, Liying Ye, Keli Yang, Xingyu Yan, Ting Gao, Fangyan Yuan, Rui Guo, Zewen Liu, Chang Li, Qiong Wu, Jiajia Zhu, Yongxiang Tian, Bo Tang, Qiqi Song, Danna Zhou

**Affiliations:** 1 Key Laboratory of Prevention and Control Agents for Animal Bacteriosis (Ministry of Agriculture and Rural Affairs), Hubei Provincial Key Laboratory of Animal Pathogenic Microbiology, Institute of Animal Husbandry and Veterinary, Hubei Academy of Agricultural Sciences, Wuhan, 430070 China; 2 GuoTai (Taizhou) Center of Technology Innovation for Veterinary Biologicals, Taizhou, 225300 China; 3 Tianjin Key Laboratory of Agricultural Animal Breeding and Healthy Husbandry, College of Animal Science and Veterinary Medicine, Tianjin Agricultural University, Tianjin, 300384 China

**Keywords:** *Mesomycoplasma* (*Mycoplasma*) *hyopneumoniae*, Mhp390, plasminogen, interaction, respiratory barrier invasion

## Abstract

The destruction of the respiratory barrier caused by *Mesomycoplasma* (*Mycoplasma*) *hyopneumoniae* plays a pivotal role in facilitating secondary infections by other respiratory pathogens. However, the pathogenesis of *M. hyopneumoniae* breaching the respiratory barrier to establish infection remains largely elusive. In this study, the role of Mhp390 encoded by *M. hyopneumoniae* in invasion of the respiratory tract barrier, including extracellular matrix and tracheal epithelial cells, were investigated through the Transwell assay. Our finding indicated that *M. hyopneumoniae* may exploit the host fibrinolytic system via Mhp390 to accumulate activated plasmin outside its membrane, thereby breaching the respiratory tract barrier and facilitating the progression of infection. Furthermore, the key functional domains within Mhp390 involved in its interaction with host plasminogen were determined by using truncated mutation techniques. Collectively, these findings will enhance our understanding of the mechanism underlying respiratory barrier invasion by *M. hyopneumoniae* thereby providing new theoretical basis for the development of novel vaccines and effective control strategies against secondary infection.

**Supplementary Information:**

The online version contains supplementary material available at 10.1186/s13567-025-01551-7.

## Introduction

*Mesomycoplasma hyopneumoniae* (*M. hyopneumoniae*, Mhp) is considered to play a primary role in porcine respiratory disease complex (PRDC) and is the primary etiological agent of porcine enzootic pneumonia (EP), a highly contagious and chronic respiratory disease that poses substantial challenges to the global swine industry [1]. Fablet et al. found that 70.8% of pigs tested positive for *M. hyopneumoniae* in 125 farrow-to-finish pig herds in France, indicating that *M. hyopneumoniae* is a predominant pathogen involved in pneumonia-like lesions [2]. The initial mycoplasmal infection is frequently complicated by secondary bacterial and viral infections, leading to more severe pulmonary lesions and increased production losses [3]. The co-infection of *M. hyopneumoniae* and porcine reproductive and respiratory syndrome virus (PRRSV) or *Actinobacillus pleuropneumoniae* (APP) can significantly exacerbate pulmonary damage and increase susceptibility to secondary infections [4–6]. Additionally, Saade et al. indicated that *M. hyopneumoniae* frequently co-infected with other bacteria or viruses to intensify the severity of the clinical disease signs [7].

The extracellular matrix (ECM) and tracheal epithelial cells serve as critical defense barriers within the respiratory system, demonstrating a robust capacity to prevent the invasion of pathogenic microorganisms such as viruses and bacteria [8]. The ECM interfaces directly with the external environment, while mucus secreted by epithelial cells efficiently entraps pathogens, dust particles, and other foreign substances [9]. These trapped pathogens are then transported upward by ciliary motion and ultimately expelled from the body. Importantly, this defensive barrier constitutes a vital protective strategy against respiratory pathogens [10]. Since Mhp invasion of the respiratory epithelium is a critical step in establishing infection, we hypothesize that inhibiting Mhp invasion of the respiratory barrier represents a novel strategy for investigating the pathogenesis and therapy of Mhp infection.

The host fibrinolytic system plays a crucial role in the degradation of the extracellular matrix [11]. Studies have shown that infection with *M. hyopneumoniae* triggers the activation of plasminogen (Plg) into plasmin, resulting in a significant increase in plasmin activity in the bronchoalveolar lavage fluid of infected pigs [12]. *Mesomycoplasma hyorhinis* (*M. hyorhinis*) can specifically bind to plasminogen via its encoded GAPDH or Eno proteins, subsequently activating it into plasmin [13, 14]. This activation accelerated the degradation of the extracellular matrix, including cilia, thereby facilitating the invasion of *M. hyorhinis* into host [13]. Seymour et al*.* revealed that *M. hyopneumoniae* employed the adhesin proteins P97 and P102 to mediate the recruitment of plasminogen onto its cell surface [15, 16]. However, whether *M. hyopneumoniae* exploits the host fibrinolytic system via other membrane proteins to accelerate the degradation of the respiratory tract barrier, including the extracellular matrix and epithelial cells, are rarely reported [17].

This study explored the role of Mhp390, encoded by *M. hyopneumoniae*, in the invasion of the respiratory tract barrier and its potential relevance to host plasmin activation. The findings revealed that anti-Mhp390 antibodies inhibited *M. hyopneumoniae* from destroying and penetrating porcine tracheal epithelial cells (PTEC). The destructive effect of rMhp390-bound plasmin on the extracellular matrix was observed using scanning electron microscopy. We also examined the key functional domains within Mhp390 that are involved in its interaction with host plasminogen.

## Materials and methods

### Bacterial strains, cultivation conditions, and DNA extraction

*Escherichia coli* strains DH5a and BL21 (DE3) were cultured at 37 °C in Luria Bertani broth or on solid medium containing kanamycin (50 mg/mL) or ampicillin (100 mg/mL). *M. hyopneumoniae* strain Hubei-1 was isolated in our lab from a pig exhibiting typical clinical signs and pathogenic characteristics of mycoplasmal pneumonia. The strain was cultured in KM_2_ cell-free medium (a modified Friis medium) containing 20% (v/v) swine serum at 37 °C [18]. The culture was harvested by centrifugation at 12 000 × *g* for 30 min from a volume of 100 mL KM_2_ cell-free medium. Subsequently, total genomic DNA was extracted from mycoplasma cells using a TIANamp Bacteria DNA Kit (Tiangen, Beijing, China, Cat No. DP302-02) following the manufacturer’s instructions.

Porcine tracheal epithelial cells (PTEC) were purchased from the Bluefbio Co., Ltd (BFB, Shanghai, China, Cat No. BFN60806663), and cultured in Dulbecco’s modified Eagle medium (DMEM, Gibco, USA, Cat No. C11965500BT) supplemented with 10% heat-inactivated fetal bovine serum (FBS, Cat No. 10099-141), 100 U/mL penicillin, and 100 mg/mL streptomycin at 37 °C in a humidified 5% CO_2_ incubator.

### Bioinformatic analysis

The HDOCK [19] and the HADDOCK [20] websites were used to predict the possible molecular docking between Mhp390 and Plg. The highest docking score was selected for docking analysis and visualized through LigPlot^+^ 2.2 (EMBL-EBI) and PyMol 3.0 (DeLano Scientific LLC). Moreover, the hydrogen bond length was confirmed through LigPlot. The antigenic determinant regions were predicted based on the Kolaskar-Tongaonkar and Emini algorithms. The secondary structure, including coils, α-helices, and β-strands within Mhp390, were predicted via Phyre2 [21]. The SignalIP and TMpred were utilized to predict the possible signal peptide and transmembrane domain of Mhp390, respectively. Multiple sequence alignment of Mhp390 amino acid was conducted using the MEGA software (version 12.0). The resulting FASTA-formatted alignment file was converted into ClustalW format using the EMBL tool [22], and then visualized in Jalview software (version 2.11). The phylogenetic tree of Mhp390 among *Mycoplasma* species was constructed based on Mhp390 amino acid sequences using the Maximum Likelihood method implemented in MEGA software (version 12.0). The lipoprotein (GenBank: CAM62957.1) of *Mycobacteroides abscessus* was used as outgroup. Subsequently, the tvBOT [23, 24] and the iTOL [25] websites were employed to annotate and visualize the phylogenetic tree.

### Primers, coding sequences amplification and recombinant plasmid construction

In order to maintain the integrity of the functional domain as much as possible, the Mhp390 protein was truncated into three segments according to the bioinformatic analysis results. The primers utilized in the current study are listed in Table 1. The recombinant plasmid pET30a-Mhp390, which was previously constructed in our study [26], served as the template. The three-truncated Mhp390 genes were amplified and cloned into the pET-30a( +) vector through *Bam*H I and *Xho* I restriction enzyme sites, followed by confirmation of nucleotide sequences through DNA sequencing utilizing the ABI3730 capillary sequencer.

**Table 1:** **Primers used for truncated rMhp390 protein construction and quantitative detection of Mhp**

Gene	Genbank Accession	Primer	Primer sequence	Restriction Enzyme
Mhp390_27-247_	AGM22196.1	Mhp390_27-247_-F^a^	5′-TTCGGATCCATGCAGAAAGAACAAGT-3′	*Bam*H I *Xho* I
		Mhp390_27-247_-R^a^	5′-TTCCTCGAGTTAGGCAAATTCAAGAG-3′	
Mhp390_248-424_	AGM22196.1	Mhp390_248-424_-F^a^	5′-CTCGGATCCATGACTAAATTTATTAACG-3′	*Bam*H I *Xho* I
		Mhp390_248-424_-R^a^	5′-CTCCTCGAGATTAATAAATTGAAACGG-3′	
Mhp390_425-604_	AGM22196.1	Mhp390_425-604_-F^a^	5′-TATGGATCCATGTATTCAGGCGGT-3′	*Bam*H I *Xho* I
		Mhp390_425-604_-R^a^	5′-CTCCTCGAGTTAATTTTGTTCATCAATTAG-3′	
Mhp494	AAV27918.1	p110-F^b^	5′-AGGATACAAACTGAGAAACCGAGCTA-3′	/
		p110-R^b^	5′-AGGTCATACCCACTCGGTCTTG-3′	

F: forward primers; R: reverse primers

^a^ primers designed for construct the recombinant plasmids

^b^ primers were used for detection of Mhp through quantitative real-time PCR

### Protein expression and purification

To preserve the integrity of functional motif within rMhp390 while removing its transmembrane regions, rMhp390 was truncated into three distinct proteins: rMhp390_27-247_, rMhp390_248-424_, and rMhp390_425-604_. The recombinant proteins Mhp390, truncated Mhp390_27-247_, truncated Mhp390_248-424_, and truncated Mhp390_425-604_ with a 6 × His-tag were expressed and subsequently purified using Ni affinity chromatography via the AKTA FPLC system (Amersham Biosciences UPC-900, Piscataway, NJ, USA). The purified proteins were confirmed by 12% SDS-PAGE gels. Protein concentrations were quantified using the BCA Protein Assay Kit (Beyotime, China, Cat No. P0010S). The ToxinEraser^™^ Endotoxin Removal Kit (Genscript, China, Cat No. L00338) was utilized for the removal of endotoxins from purified proteins in accordance with the manufacturer's instructions. Finally, the proteins were stored at -80 ℃ for future use.

### Preparation and characterization of monoclonal antibody against Mhp390

The mice utilized in this study were procured from the Hubei Experimental Animal Research Center. All animal experiments were carried out in strict adherence to the International Guiding Principles for Biomedical Research Involving Animals (1985) and were approved by the Ethics Committee of the Institute of Animal Husbandry and Veterinary, Hubei Academy of Agricultural Sciences (Wuhan, China; identification code: SCXK(E)2023-0618, date of approval: 20,230,712). Immunization of BALB/c mice was performed by injecting the purified rMhp390 protein (100 μg) emulsified with complete (the first immunization) and incomplete (the second and third immunization) Freund’s adjuvant (1:1, v/v) into the neck and back of the mice subcutaneously to produce monoclonal antibody. The interval between the second immunization and the first immunization is two weeks, while the third and the second immunization is three weeks. One day after the last boost with 100 μg of the purified rMhp390 protein, the splenocytes of the immunized BALB/c mice were fused with SP2/0 myeloma cells using polyethylene glycol (PEG solution, Sigma, Germany, Cat No. P7181). The hybridomas were selected through selective HAT medium (Sigma, Germany, Cat No. H0262).

The positive clones were identified by indirect enzyme linked immunosorbent assay (iELISA) utilizing the rMhp390 protein (coating concentration at 0.5 μg/mL), while a negative control (BSA, 0.5 μg/mL) was employed for comparison. After undergoing four rounds of subcloning, hybridomas capable of producing monoclonal antibodies (mAbs) were successfully established and characterized through iELISA and chromosome analysis. Mice ascites were generated by injecting the hybridomas into the paraffine primed BALB/c mice. After purification of ascites fluid through the IgG subclass mouse monoclonal antibody purification kit (Beijing Biodragon, China, Cat No. BF06190), the mouse monoclonal antibody subtype identification kit (Proteintech, USA, Cat No. PK20003) was used for mAb isotyping according to the manufacturer’s instructions. Western Blotting and iELISA assay were performed to determine the reactivity and specificity of the prepared monoclonal antibody. Purified monoclonal antibody was stored at −80 °C until use.

### Immunogenicity analysis of Mhp390

Microtiter plates were coated with 0.4 μg/well of Mhp390 protein in 100 μL carbonate-bicarbonate buffer (pH 9.8) at 4 °C overnight. The plates were washed with PBST and subsequently incubated with a blocking buffer (5% non-fat dry milk) at 37 °C for 2 h. After washing, the wells were exposed to serum (100 μL, diluted in a ratio of 1:40) from pigs naturally infected with *M. hyopneumoniae* or immunized with inactivated vaccine (Boehringer Ingelheim). The serum from healthy pig was served as the negative control. After further washing, horseradish peroxidase (HRP)-conjugated goat anti-pig IgG (Biodragon, China, Cat BF03030) was added to each well and incubated at 37 °C for 1 h. After washing with PBST, the wells were subsequently exposed to TMB chromogen solution (Beyotime, China, Cat No. P0209) and stopped with stop solution (Beyotime, China, Cat No. P0215). The absorbance was measured at OD_450nm_ using a microplate reader. Triplicate samples were used to calculate mean and standard deviation (S.D.) values.

### Interaction between rMhp390 and host plasminogen

Specific-pathogen-free pigs (4–6 weeks old), free of *M. hyopneumoniae*, porcine circovirus 2, and porcine reproductive and respiratory syndrome virus, were procured from a herd maintained at the Hubei Academy of Agricultural Sciences. The fresh blood with anticoagulant was collected and the plasma was obtained through centrifugation. The mixture of 100 mL lysine Sepharose 4B (Solarbio, China, Cat No. S8911) and 300 mL plasma was stirred at 4 °C for 5 h. Subsequently, the mixture was added to the affinity column and filtered using phosphate buffer (PBS, pH = 8.0, 0.1 mol/L) at 4 °C until the OD_280nm_ reached less than 0.1. The washing process was continued at 10 °C using PBS (pH = 8.0, 0.3 mol/L) until the OD_280nm_ value dropped below 0.02. The temperature was reduced to 4 °C. Following balance with PBS (pH = 8.0, 0.1 mol/L), elution was conducted using 0.015 mol/L 6-aminocaproic acid (ε-ACA, Sigma, Germany, Cat No. A7824) in PBS (pH = 8.0, 0.1 mol/L). The protein peak was detected at OD_280nm_, followed by collection of the active fraction. Subsequently, the affinity column was reloaded and a linear gradient elution using 0–0.015 mol/L ε-ACA was employed to collect the protein peak. The protein was purified and concentrated via ultrafiltration tube (MWCO 30 kDa, Millipore, USA, Cat No. UFC903008).

The binding activity of rMhp390 to host plasminogen was evaluated using ELISA and BLI assays. In brief, microtiter plates were coated with 0.8 μg/well of the Plg in 50 mM carbonate-bicarbonate buffer (pH = 9.8) at 4 °C overnight. The plates were washed with PBST (PBS containing 0.01% (V/V) Tween-20) and subsequently incubated with a blocking buffer consisting of non-fat dry milk at a concentration of 5%, maintained at a temperature of 37 ℃ for a duration of 2 h. After washing with PBST, the wells were exposed to 100 μL rMhp390, truncated rMhp390_27-247_, truncated rMhp390_248-424_, and truncated rMhp390_425-604_ proteins at concentration of 1 μg/mL, respectively. Purified rP97R1 protein (1 μg/mL) served as the positive control [27], while the purified host NOD1 protein (1 μg/mL) served as the negative control [28]. The plates were washed with PBST to eliminate non-adherent proteins, followed by the addition of a His-tag monoclonal antibody (Proteintech, USA, Cat No. 66005) into each well and incubation at 37 °C for 60 min. After further washing with PBST, horseradish peroxidase (HRP)-conjugated goat anti-mouse antibody (Proteintech, USA, Cat No. SA00001) was added to the wells and incubated at 37 °C for 60 min. After washing with PBST, the wells were subsequently exposed to TMB chromogen solution (Beyotime, China, Cat No. P0209) and stopped with stop solution (Beyotime, China, Cat No. P0215). The adherence activity was assessed by measuring the optical density values at OD_450nm_ using an automated microplate reader. Data were represented as mean ± SD from three independent replicates.

The biolayer interferometry (BLI) were performed to monitor binding kinetics of rMhp390 and Plg through a ForteBio Octet Red96 instrument (ForteBio, USA). Briefly, the Plg protein was biotinylated via a biotin labeling kit (Genemore, China, Cat No. G-MM-IGT) and subsequently immobilized onto streptavidin (SA) biosensors. After a 240 s balance in PBST buffer (PBS containing 0.02% (v/v) Tween20), associations between rMhp390 (different protein concentrations or different truncated segments) and Plg (25 μg) were performed for 300 s. Dissociation process was conducted in PBST buffer for a duration of 150 s. The kinetic dissociation rate constant (K_D_) was determined utilizing a 1:1 binding model. The Data Analysis HT software (ForteBio, USA) was employed to determine the association and dissociation constants.

### rMhp390 accelerates the degradation of PTECs and ECM

The ability of the resulting rMhp390-bound plasmin to degrade the confluent PTEC cell layer was investigated using a Transwell assay. PTEC cells were propagated in DMEM supplemented with 10% FBS on the 3.0 μm transparent membrane inserts placed in 6-well plates (Labselect, China, Cat No. 14122). The cultivation of *M. hyopneumoniae* took place in a 5% CO_2_ atmosphere at 37 °C for a duration of 42 h. The washed *M. hyopneumoniae* (10^8^ CCU) was suspended in 1 mL PBS containing anti-Mhp390 mAb (diluted 1:100) and incubated at 37 °C for 30 min. Subsequently, the mAb treated Mhp was added to the upper compartment of the transwell, where PTEC cells (10^6^) were cultured in DMEM containing 10% FBS. The culture chambers were incubated at 37 °C for 45 min. The mAb untreated Mhp served as positive control. The samples from the lower compartment of the transwell were obtained and subjected to color changing unit (CCU) assay. Moreover, an equal amount liquid from the lower compartment of the transwell in each group was taken and subjected to quantitative real-time PCR assay (qPCR). The primers used for detection of relative number of *M. hyopneumoniae* that penetrated through PTEC cells were listed in Table 1.

PTEC cells were propagated in DMEM on microscope coverslips placed in 6-well plates (Corning, Inc., USA). The proteins were immobilized by using polystyrene beads (Sigma, Germany, Cat No. LB11). A total of 20 μL beads were incubated with 1 mL of rMhp390 (1 mg/mL) at 4 °C overnight. The beads were washed with PBS and subsequently incubated with bovine serum albumin (BSA, 5% weight/volume in PBS) at 37 °C for 1 h. Plg was added at a concentration of 10 μg/mL and incubated at 37 °C for 2.5 h. Subsequently, tissue plasminogen activator (tPA) was added at a concentration of 200 ng/mL and incubated at 37 °C for another 2.5 h. PBS washing was performed between each step. After washing, beads were resuspended in PBS and added to PTEC cells in 6-well plates (70 μL per well) at 37 °C for a duration of 6–9 h. Beads incubated with Mhp served as the positive control, while the beads incubated with BSA served as the negative control. The degradation of PTECs was visualized through Olympus microscope.

The degradation of commercial ECM (Matrigel, Corning, USA) by the resulting rMhp390-bound plasmin was evaluated using scanning electron microscopy (SEM). The matrigel was initially diluted in pre-chilled PBS at a ratio of 1:3. Subsequently, the diluted matrigel was added onto the 3.0 μm transparent membrane inserts (Labselect, China, Cat No. 14122). The ECM underwent gelation following a 30 min incubation at 4 °C. After gelation, the ECM was dried overnight at 37 °C. The rMhp or BSA was adsorbed onto the beads passively in accordance with the manufacturer’s instructions. Briefly, a total of 20 μL beads were suspended in 1 mL of rMhp or BSA solution (1.5 mg/mL) and incubated overnight at 4 °C. The beads were washed with PBS and subsequently incubated with BSA (5% weight/volume in PBS). Subsequently, the beads were incubated with Plg at a concentration of 10 μg/mL and incubated at 37 °C for 2.5 h. PBS washing was performed between each step to eliminate unbound protein. After incubation with tPA (500 ng/mL) for an additional 2.5 h at 37 °C, the beads were thoroughly washed and resuspended in 1 mL PBS. A volume of 70 μL of resuspended beads was added to each upper compartment of the transwell, while the lower compartment was filled with 700 μL of PBS. The culture chambers were incubated at 37 °C for a duration of 40 h. The transwell membrane was obtained and fixed with 2.5% glutaraldehyde. The degradation of ECM was visualized through scanning electron microscopy with a HITACHI S-4800 instrument (Hitachi, Japan).

### Statistical analysis

The statistical analysis was performed using the student’s *t*-test to assess potential differences among different groups. The data were presented as mean ± standard deviation (SD). A *P*-value < 0.05 was considered statistically significant, while a *P*-value < 0.01 was considered highly significant.

## Results

### Sequence characterization and immunogenicity analysis of Mhp390

To investigate the sequence divergence of Mhp390, a multiple alignment of the amino acid sequences of the Mhp390 was conducted among *Mycoplasma* species (Additional file 1). The amino acid sequence of Mhp390 is highly conserved among *M. hyopneumoniae* (with a sequence similarity of over 98%), and there is only one copy of Mhp390 in the genome of *M. hyopneumoniae*. Interesting, three homologs of the Mhp390 related gene were observed in the genomes of *M. hyorhinis*, *M. bovis*, *M. conjunctivae*, and *M. ovipneumoniae*; while two homologs were found in the genomes of *M. collis* and *M. neurolyticum*. The Mhp390 gene was not detected in the genomes of *M. hyosynoviae*, *M. genitalium*, *M. moatsii*, or *M. mobile*, whereas these strains primarily infect non-respiratory tissues, including the intestinal tract, joints, and genital tract. In addition, a phylogenetic tree of the distribution and genetic distance of Mhp390 protein among *Mycoplasma* species was established (Additional file 2), in which the lipoprotein of *Mycobacteroides abscessus* was used as outgroup.

The presence of anti-Mhp390 antibodies in serum from pigs naturally infected with *M. hyopneumoniae* or immunized with inactivated vaccine (Boehringer Ingelheim) were investigated. As shown in Additional file 3, both serum from pigs immunized with the inactivated vaccine and naturally infected serum could react with the rMhp390 protein, but no obvious reaction was observed with healthy swine serum.

### Production and purification of monoclonal antibody against Mhp390

BALB/c mice were immunized with purified rMhp390 protein to produce monoclonal antibody. After four subclonings, hybridomas producing mAbs were established and sent to the Chinese Typical Culture Preservation Center for storage (CCTCC NO: C2024161). The hybridoma cell line exhibited stability throughout continuous passage, with an average chromosomal count of 112.8. Moreover, the secreted monoclonal antibody displayed a heavy chain subtype of IgG2b and a light chain subtype of Kappa. The ELISA and indirect immunofluorescence assay, established by utilizing this monoclonal antibody, exhibit high specificity in *M. hyopneumoniae* detection (Additional file 4). The developed ELISA assay is capable of detecting a minimum concentration of total *M. hyopneumoniae* antigen at 2.5 ng/mL (data not shown).

### The anti-Mhp390 antibody blocks *M. hyopneumoniae* from penetrating PTEC cells

**Figure 1:**
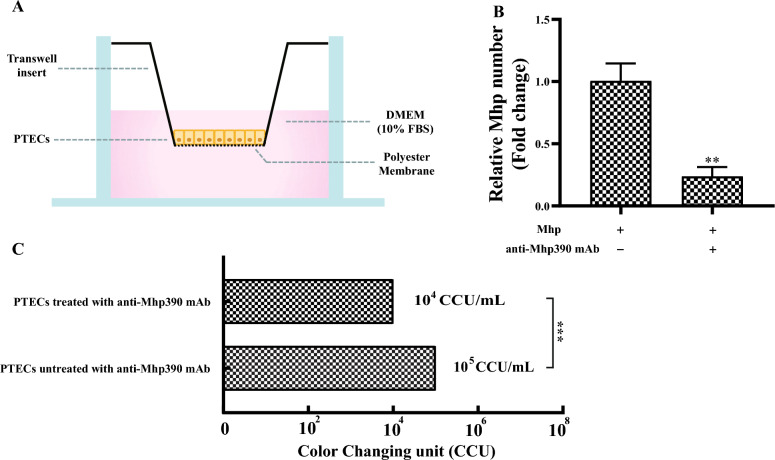
**Relative quantity of *M. hyopneumoniae* penetrating through PTECs**. **A** Schematic representation of the Transwell assay utilized for investigating *M. hyopneumoniae* penetration through PTECs. **B** The relative number of Mhp that penetrated through PTEC cells was determined through qPCR. **C** The relative quantity of Mhp that penetrated through PTEC cells was determined through CCU assay. The Mhp penetrated through PTEC cells treated with anti-Mhp390 mAb was 10^4^ CCU/mL. The Mhp penetrated through PTEC cells untreated with anti-Mhp390 mAb was 10^5^ CCU/mL. CCU: color changing unit, DMEM: Dulbecco’s modified Eagle medium, FBS: fetal bovine serum, PTEC: porcine tracheal epithelial cells. **P < 0.01, and ***P < 0.001 indicate statistically significant differences among different groups.

The role of Mhp390 in *M. hyopneumoniae* invasion of PTEC cell layer was examined by determining the penetration blocking effect of the anti-Mhp390 antibody. As shown in Figure 1C, the CCU assay revealed a higher quantity of *M. hyopneumoniae* (10^5^ CCU/mL) penetrating the PTEC layer compared to the Mhp390-antibody blocking group (10^4^ CCU/mL). As expected, the quantitative real-time PCR result was consistent with the CCU assay, demonstrating a decrease in *M. hyopneumoniae* penetration of PTEC cell barrier following treatment with Mhp390-antibody (Figure 1B).

### Degradation of ECM by the rMhp390 bound plasmin

**Figure 2:**
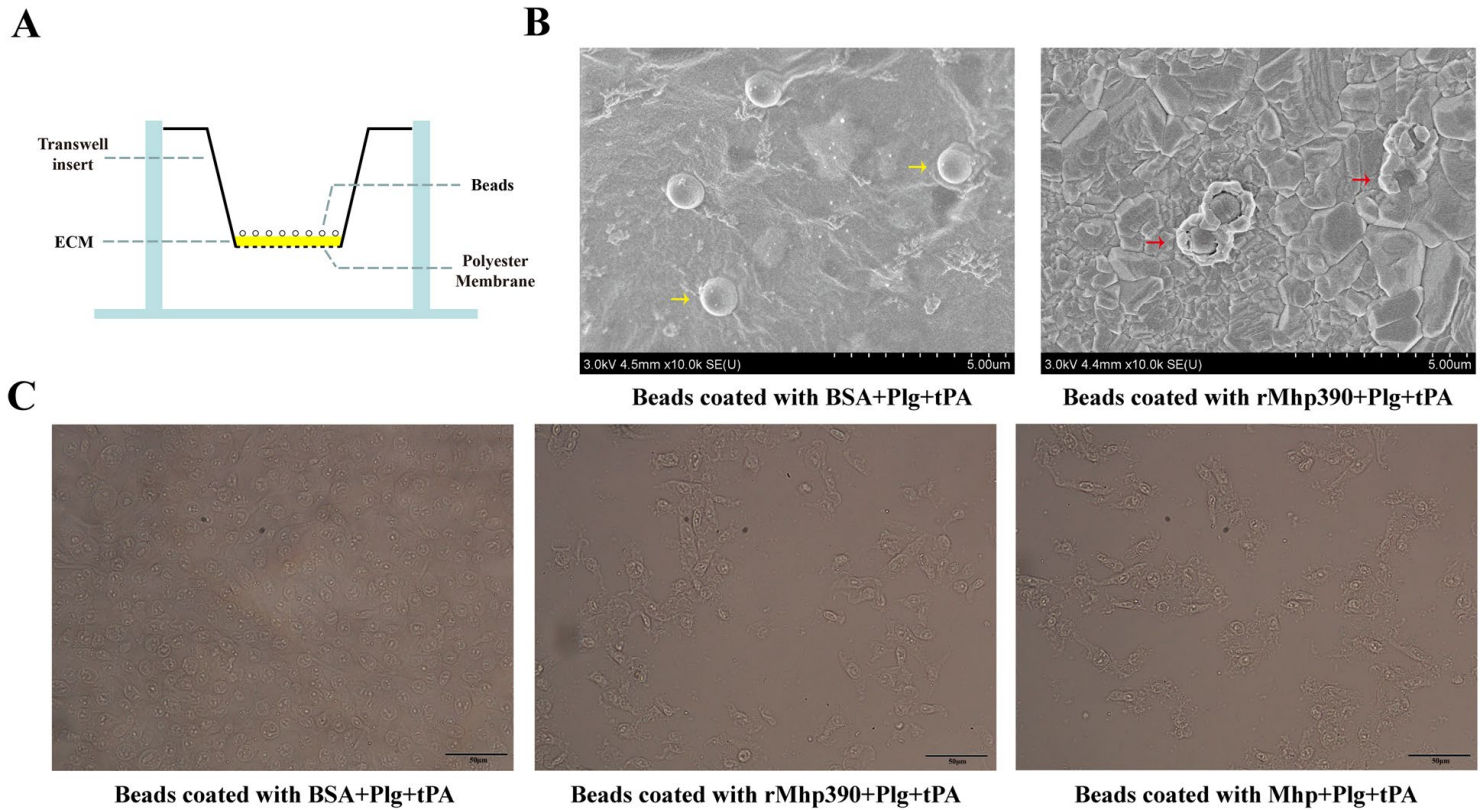
**rMhp390 accelerates the degradation of PTECs and ECM.** **A** Schematic illustration for investigating the degradation of extracellular matrix by rMhp390-bound plasmin. Matrigel was reconstituted on 3.0 μm transparent membrane inserts. The rMhp or BSA was passively adsorbed onto the beads, which were subsequently treated with Plg and tPA. The capacity of rMhp390-bound plasmin to degrade extracellular matrix was then analyzed. **B** The degradation of extracellular matrix induced by the rMhp390-bound plasmin was visualized by scanning electron microscopy. **C** The degradation of PTECs induced by the rMhp390-bound plasmin was assessed through Olympus microscope. BSA: bovine serum albumin, ECM: extracellular matrix, Plg: plasminogen, tPA: tissue plasminogen activator.

The destructive effects of rMhp390 on the extracellular matrix was investigated through scanning electron microscopy. As shown in Figure 2B, the ECM remains intact after treatment with BSA-incubated beads, without apparent compromise in its integrity. Upon treatment with rMhp390-incubated beads, the ECM surrounding the beads exhibited evidence of damage, including both surface damage and penetration, indicating potential penetration of the ECM by rMhp390-incubated beads. These findings revealed that Mhp390, encoded by *M. hyopneumoniae*, contributes to the degradation of extracellular matrix.

### Degradation of PTECs by the rMhp390 bound plasmin

We next examined whether the degradation of the PTEC cells by *M. hyopneumoniae* was relevant to Mhp390 bound plasmin. The destructive effects of rMhp390 on respiratory epithelial cells was investigated through a confocal microscope. Notably, PTEC cells undergo extensive degradation when exposed to beads incubated with rMhp390 and Mhp, while no obvious cell degradation was observed when beads inoculated with BSA (Figure 2C). The findings demonstrated that *M. hyopneumoniae* utilizes Mhp390 to recruit Plg and activate plasmin, thereby facilitating its penetration through the PTEC cell barrier.

### Binding activity of rMhp390 to host plasminogen

**Figure 3:**
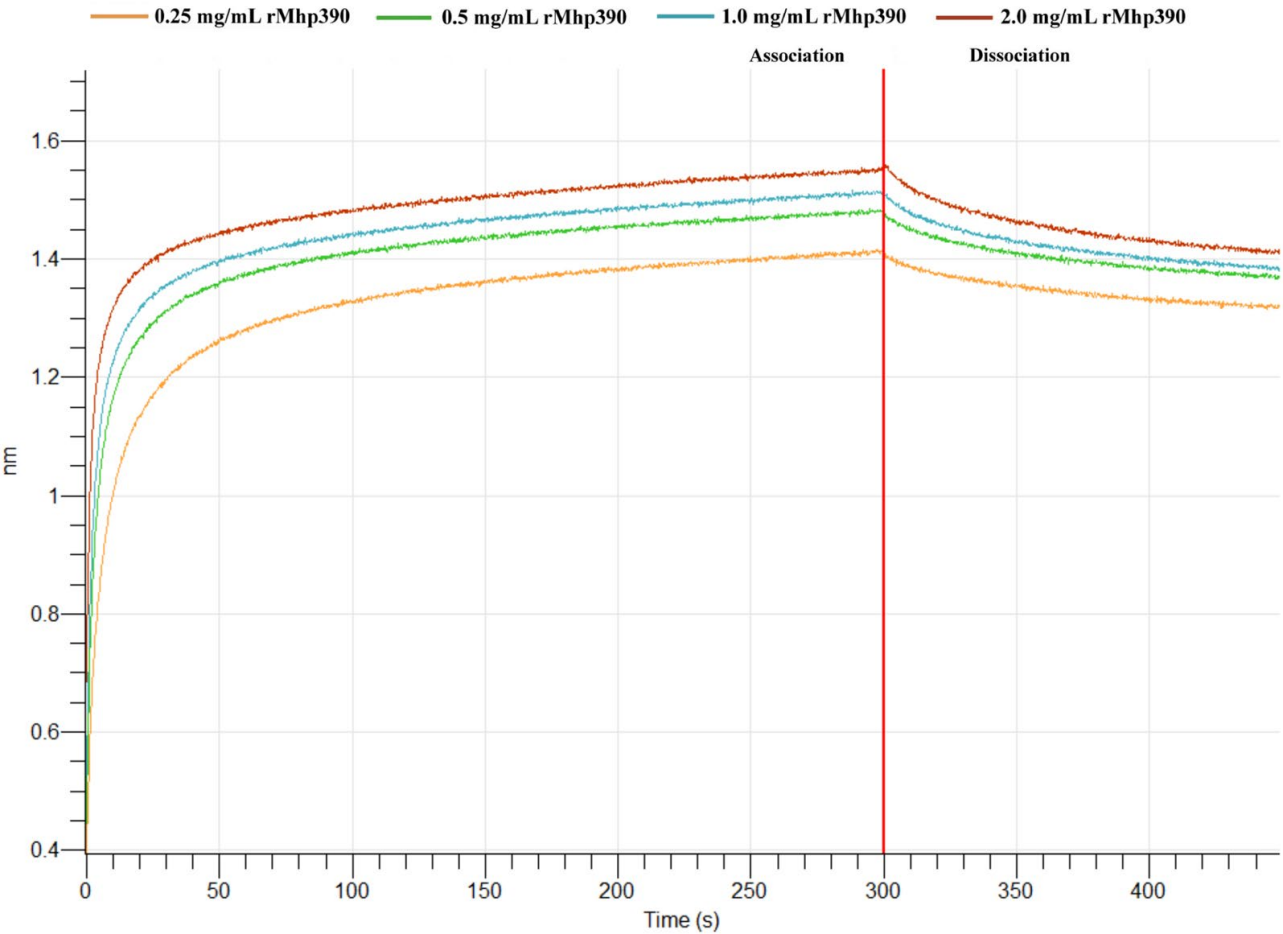
**Kinetic analysis of the affinity between Plg and rMhp390 (various concentrations) through bio-layer interferometry**. The binding kinetics of rMhp390 to Plg was revealed by BLI assay. The left side of the red line indicates the association kinetics of rMhp390 with Plg, while the right side indicates the dissociation process. rMhp390 was rapidly bound to but slowly dissociated from SA-biosensors coated with Plg.

The binding kinetics of rMhp390 to host plasminogen was revealed by BLI assay (Figure 3). Binding curves were generated with five different concentrations of rMhp390 and showed a dose-dependent increase in signals. As shown in the association and dissociation kinetic chart, rMhp390 was rapidly bound to but slowly dissociated from SA-biosensors coated with Plg. The curves from the low and high concentrations showed the same behavior, higher concentrations showed steeper increase in the signal during association.

### The potential functional domains involved in the interaction between Mhp390 and Plg

**Figure 4:**
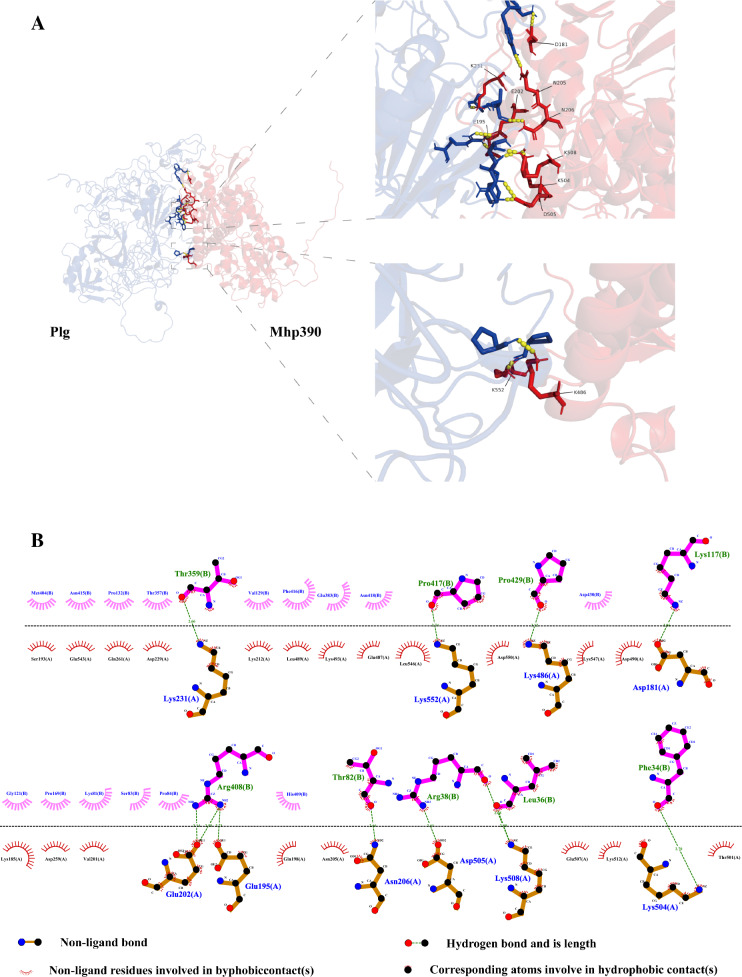
**Analysis of potential domains involved in the interaction between Mhp390 and Plg.** **A** The potential molecular docking involved in the interaction between Mhp390 and Plg. **B** The hydrogen bond lengths at the docking site predicted via LigPlot. The amino acid residues within Mhp390 that potentially contribute to the formation of hydrogen bonds are indicated below the horizontal dotted line, while those within Plg are indicated above this line.

The antigenic determinant regions were predicted based on the Kolaskar-Tongaonkar and Emini algorithms, resulting in the identification of four segments with the highest scores: P69-N80, D103-L113, G134-N147, and I503-S513 (Additional file 5A), respectively. More importantly, the potential molecular docking involved in the interaction between Mhp390 and Plg were predicted through the HDOCK and HADDOCK analysis, and visualized through LigPlot and PyMol program (Figure 4A). As shown in Figure 4B, the residues D181, E195, E202, N206, K231, K486, D505, K508, and K552 exhibited potential for hydrogen bond formation between rMhp390 and Plg. The hydrogen bond lengths at the docking site were determined using LigPlot and arranged in descending order of their binding affinity, as depicted in Figure 4B. The secondary structure, including coils, α-helices, and β-strands within Mhp390, were predicted via Phyre2 (Additional file 5B). The SignalIP and TMpred were utilized to predict the possible signal peptide and transmembrane domain of Mhp390, respectively. The cleavage site of the signal peptide was identified between residues 26aa and 27aa, while the transmembrane helix spanned residues 9aa to 25aa in N-terminal region.

### Expression and purification of rMhp390 and its truncated proteins

**Figure 5:**
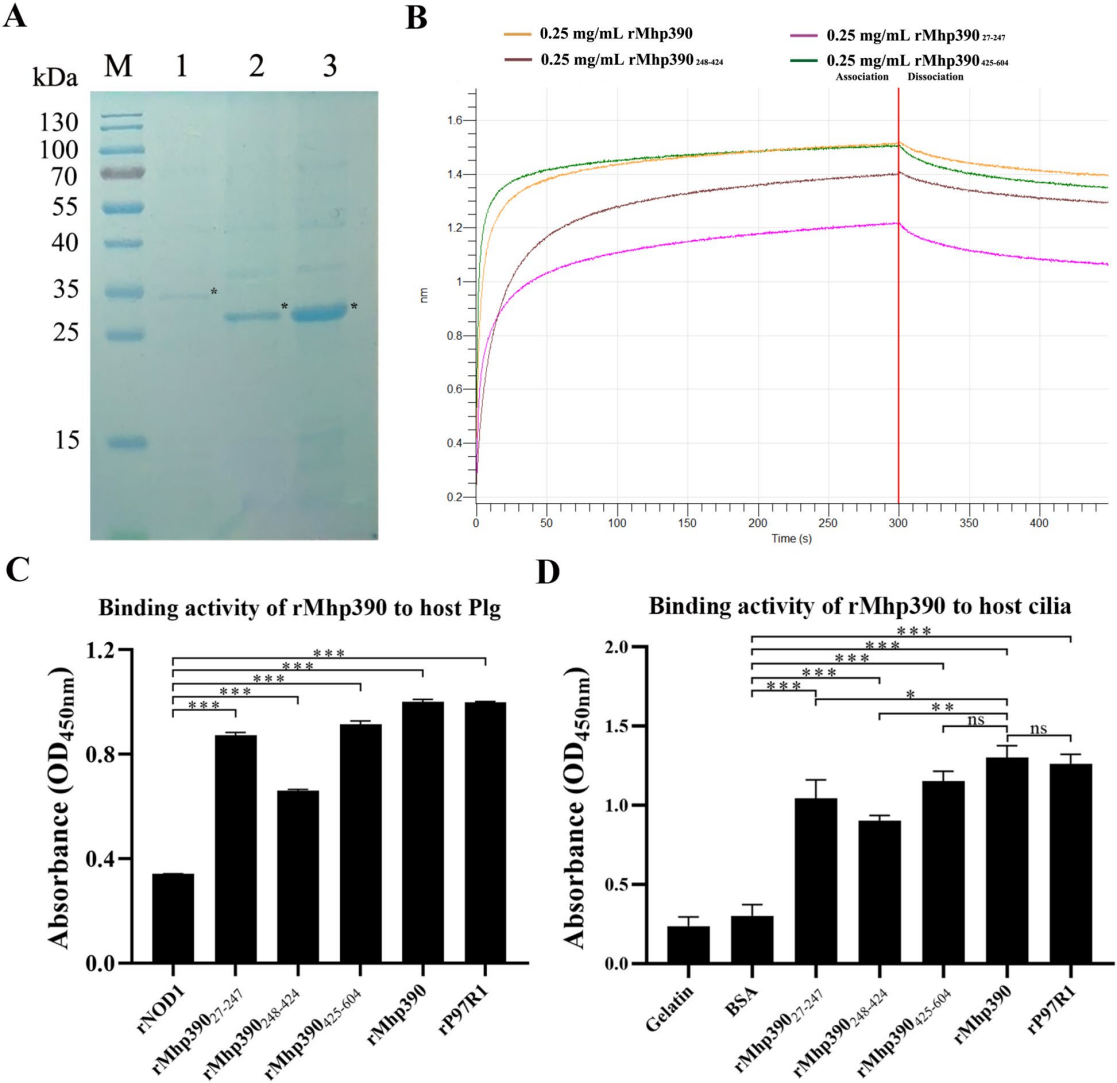
The functional domains involved in the interaction between Mhp390 and Plg. **A** The purified proteins of truncated rMhp390. M, Protein Marker (kDa); lane 1, purified rMhp390_27-247_ by Ni–NTA column; lane 2, purified rMhp390_248-424_ by Ni–NTA column; lane 3, purified rMhp390_425-604_ by Ni–NTA column. **B** Kinetic analysis of the affinity between truncated rMhp390 and Plg through bio-layer interferometry. **C** Binding activity of truncated rMhp390 to Plg. **D** Binding activity of truncated rMhp390 to cilia. *P < 0.05, **P < 0.01, and ***P < 0.001 indicate statistically significant differences among different groups, and ns represents no difference.

Based on bioinformatics analysis, the potential binding domains between rMhp390 and Plg are primarily spanned residues regions 181aa-231aa and 486aa-552aa. rMhp390 was truncated into three distinct proteins: rMhp390_27-247_, rMhp390_248-424_, and rMhp390_425-604_. As shown in Figure 5A, these three truncated rMhp390 proteins were detected by SDS-PAGE as bands at approximately 30 kDa, 27 kDa, and 27 kDa, respectively. Each band corresponds to the size with a His tag, indicating the correct expression of the truncated proteins. The concentration of the purified protein was measured by using the BCA Protein Assay Kit. The endotoxin in each purified protein and Plg was eliminated using the ToxinEraser^™^ Endotoxin Removal Kit, following the manufacturer’s instructions.

### Interaction between rMhp390 and host plasminogen

The microtiter plate-based adherence assay was utilized to further assess the binding activity of rMhp390 to host plasminogen (Figure 5C). Notably, the results demonstrated that rMhp390 exhibited highest level of binding activity with Plg, followed by P97R1. There was no statistical difference in the binding activity of Plg to rMhp390 or P97R1. Among truncated proteins group, rMhp390_27-247_ showed high binding activity to Plg (2.56-fold), albeit a slightly lower than that of rMhp390_425-604_ (2.68-fold), in comparison to negative control wells coated with rNOD1 (*P* < 0.001). Moreover, the binding capacity of three truncated proteins to cilia was also measured. As shown in Figure 5D, the rMhp390_425-604_ exhibited highest binding activity with cilia, which is superior to that of rMhp390_27-247._

To gain more insights into the interaction between different domains of rMhp390 and Plg, we immobilized Plg on biosensors and analyzed its interaction with three truncated rMhp390 proteins through biolayer interferometry, respectively. As shown in the association and dissociation kinetic chart (Figure 5B) the intact rMhp390 protein was rapidly bound to but slowly dissociated from SA-biosensors coated with Plg. In addition, all three truncated rMhp390 proteins exhibited binding activity with plasminogen (Figure 5B).

## Discussion

*M. hyopneumoniae* infection is frequently accompanied by co-infection with other respiratory pathogens, and this co-infection phenomenon is widely observed [4]. Previous studies have primarily focused on the impairment of ciliary function in the respiratory tract following infection with *M. hyopneumoniae*, resulting in a diminished capacity of cilia to trap and clear foreign pathogens [17, 27]. Apart from this reason, other mechanisms leading to secondary infection remain inadequately understood and warrant further exploration.

Through long-term evolution, *Mycoplasma* has undergone a process of extensive genome reduction and is recognized as the smallest self-replicating microorganisms [29]. With minimal genomic redundancy, *Mycoplasma* exploit certain proteins to perform multiple functions [30]. In a previous study, it was observed that the expression of Mhp390 was significantly upregulated by 3.25-fold in *M. hyopneumoniae* strain 168 compared to its attenuated counterpart [31]. Consequently, a functional investigation of Mhp390 was undertaken to delineate its biological functions [26]. Interestingly, Mhp390 was characterized as a cilia adhesin that plays an important role in binding to swine tracheal cilia [26]. The adhesion of *M. hyopneumoniae* to host alveolar macrophages was effectively inhibited by anti-Mhp390 serum and recombinant Mhp390 protein in a dose-dependent manner, respectively [28]. This present study investigated the interaction between *M. hyopneumoniae* and the host by examining the protein functions of *M. hyopneumoniae*-encoded Mhp390, aiming to explore the mechanisms by which *M. hyopneumoniae* compromises the respiratory tract barrier.

Plasmin, which is derived from its inactive precursor plasminogen, functions as a serine protease that degrades fibrin and various extracellular matrix (ECM) proteins, thereby disrupting the ECM barrier [32]. Bacterial pathogens have evolved diverse mechanisms to exploit the host's plasminogen/plasmin system, thereby facilitating their dissemination across tissue barriers [33]. *Yersinia pestis* ingeniously employs specialized plasminogen activators to hijack the host's plasminogen/plasmin system [34]. In contrast, the majority of pathogens anchor plasminogen to their surface through plasminogen receptors [35]. For instance, *M. hyorhinis* has the capability to capture plasminogen, which can subsequently be activated by tPA on its surface [13]. Notably, the binding activity of rMhp390 to host plasminogen was revealed by both BLI (Figure 3) and microtiter plate-based adherence assay (Figure 5C). These results provide evidence that the surface located Mhp390 is the plasminogen receptor of *M. hyopneumoniae*. As shown in Figure 1, a decrease in *M. hyopneumoniae* penetration of PTEC cell barrier was found following treatment with Mhp390-antibody, suggesting that *M. hyopneumoniae* is likely to exploit Mhp390 to invade the barrier of porcine tracheal epithelial cells. In addition, the destructive effects of rMhp390 on the extracellular matrix was confirmed through scanning electron microscopy. The capacity to degrade tissue barriers formed by the extracellular matrix, resulting in damage to tissues and structures, is a critical determinant of bacterial invasiveness within the host.

The binding activity of rMhp390 to host plasminogen or cilia was assessed through microtiter plate-based adherence assay. Among truncated rMhp390 proteins, the rMhp390_425-604_ exhibited highest level of binding activity with Plg, followed by rMhp390_27-247_. Moreover, the rMhp390_425-604_ exhibited highest binding activity with cilia, surpassing that of rMhp390_27-247_. These findings suggested that the functional region within the rMhp390 protein that interacts with Plg or cilia is primarily located within the residue region spanning from 425 to 604aa. In addition, no statistical difference in the binding activity of rMhp390 to host plasminogen or cilia was observed, in comparison to P97R1 (*P* > 0.05). Blastp alignment (E-value = 1e^−4^) revealed no significant sequence similarity between Mhp390 and P97, suggesting that Mhp390 and P97 may employ distinct mechanisms to bind plasminogen or cilia. It has been reported that lysine residues play a crucial role in the binding of plasminogen to enolase or DnaK from *Neisseria meningitidis* [36]. Wang et al. reported that the simultaneous mutation of Lys451 and Lys452 reduced the binding affinity of *M. hyorhinis* enolase for plasminogen, suggesting an important role of these lysines residues in the interaction [14]. Similarly, another research demonstrated that the binding of Group A *Streptococcus* enolase to human plasminogen is facilitated by two internal lysine residues at positions 252 and 255 [37]. These findings highlight the role of lysine residues in plasminogen binding. According to the hydrogen bond analysis within the docking site (Figure 4B), four lysine residues (Lys486, Lys504, Lys508, and Lys552) and one asparticaid residue (Asp505) were predicted in rMhp390_425-604_ that contribute to the formation of hydrogen bonds, while one lysine residue (Lys231), two glutamicacid residues (Glu159, Glu202), one asparticaid residue (Asp181), and one asparagine residue (Asn206) were identified in rMhp390_27-247_. No hydrogen bond within the docking site was found in rMhp390_248-424_. We speculated that higher lysine content may account for the enhanced binding affinity of rMhp390_425-604_ with Plg. Further experiments are necessary to identify the specific key amino acid residues within rMhp390_425-604_ that form hydrogen bonds with Plg during docking. In addition, Mhp390 and its truncated proteins were expressed using the *E. coli* system. This prokaryotic expression approach might alter the protein conformation compared to the native proteins produced in *M. hyopneumoniae*, thereby potentially obscuring authentic interaction details.

Pieters et al. [38] assessed the duration of *M. hyopneumoniae* infection and demonstrated that this pathogen persisted for an impressive duration of up to 214 days post-infection within the respiratory tracts of infected swine. At this time point, asymptomatic carriers retained the capability to infect susceptible animals [38]. The tenacious persistence of *M. hyopneumoniae* infection poses a threat to its prevention and control. The complement system efficiently recognizes and eradicates bacteria and viruses, serving as a crucial component of the host's defense against pathogen infection [39]. Plasminogen, upon activation to plasmin, acts as a negative regulator of the complement system [40]. Complement associated proteins, including C3b, can form covalent bonds on the surfaces of bacteria and viruses, thereby enhancing the recognition and phagocytosis of these pathogens by phagocytes such as macrophages and neutrophils [41, 42]. C5 is a key component in the complement system, and C5b serves as the initiating factor for the assembly of the membrane attack complex (MAC) [43]. The degradation of C5b effectively inhibits the formation of MAC [44]. Plasminogen interacts with complement components C3, C3b, C3d, and C5, and upon activation to plasmin, it directly cleaves C3b and C5 [45]. This cleavage inactivates these components, thereby inhibiting the function of the complement system [33]. Moreover, plasmin is capable of cleaving the hinge region of IgG antibodies that is attached to bacteria, and the removal of the Fc fragment consequently results in diminished phagocytosis by macrophages [46]. Hence, the rMhp390-bound plasmin may function as a complement inhibitor, thereby facilitating the evasion of *M. hyopneumoniae* from complement-mediated innate immune clearance. However, further experiments are required to investigate whether Mhp390 is contributes to the persistent infection of *M. hyopneumoniae*.

The surface localization and multifunctional properties of Mhp390 in *M. hyopneumoniae* pathogenesis render it a potential candidate for the development of subunit vaccines against *M. hyopneumoniae* infection [26]. Interestingly, a recombinant chimera vaccine composed of *M. hyopneumoniae* antigens P97R1, Mhp390, and P46 induced significant cellular immunologic responses (high level of IFN-γ, CD4^+^, and CD8^+^ T lymphocytes) and high production of IgG and IgM antibodies [47].

**Figure 6:**
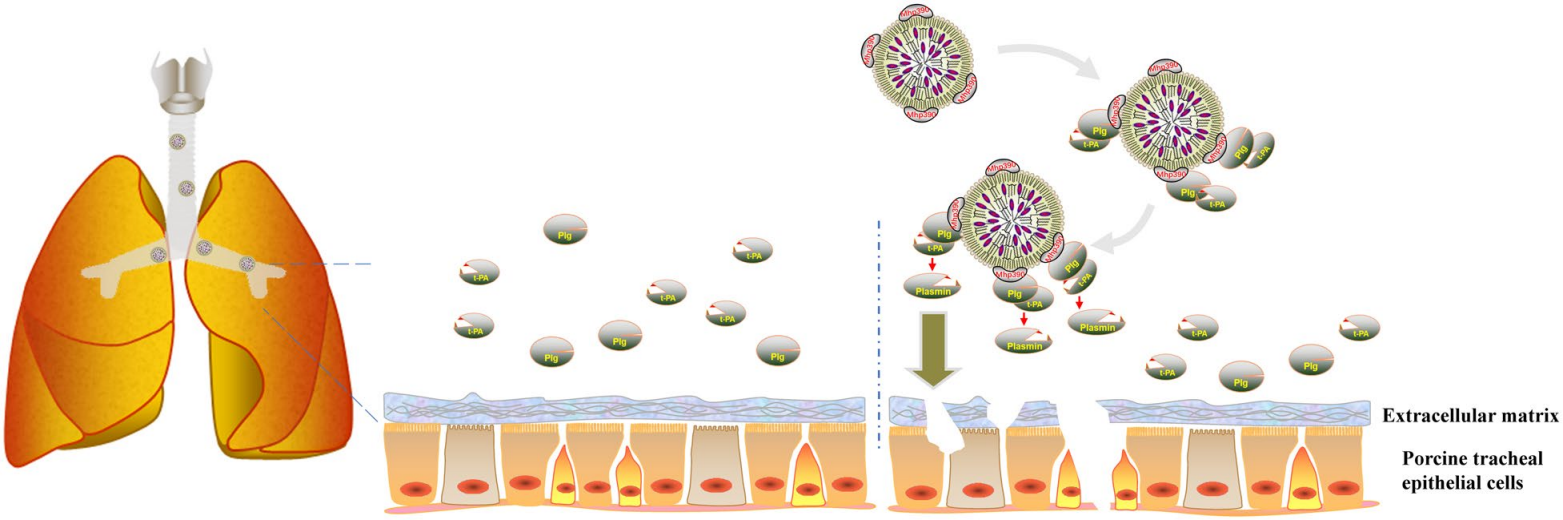
**Schematic illustration of** ***M. hyopneumoniae*** **utilizing Mhp390 for theinvasion of extracellular matrix and respiratory epithelial cells.**

Plasminogen and tPA are extensively distributed in the respiratory tract, lungs, and circulatory system of body fluids, and do not exhibit fibrinolytic activity under normal conditions [48]. As illustrated in Figure 6, we hypothesize that *M. hyopneumoniae* initially employs adhesion factors (such as P97, P102, Mhp390, etc*.*) to bind to cilia, thereby facilitating its adhesion and host colonization. Subsequently, *M. hyopneumoniae* recruits Plg and tPA to the outer surface of its membrane via Mhp390, which then activates Plg into plasmin. The Mhp390-bound plasmin leads to the degradation of extracellular matrices and PTEC cells, thereby enabling the pathogen to penetrate tissue barriers and disseminate.

To the best of our knowledge, this study provides the first evidence of plasmin-mediated degradation of reconstituted extracellular matrix and epithelial cells in the presence of *M. hyopneumoniae* Mhp390. In summary, *M. hyopneumoniae* employed lipoprotein Mhp390 to interact with host plasminogen. The destructive effects of Mhp390-bound plasmin on the extracellular matrix were confirmed via scanning electron microscopy. Interestingly, the anti-Mhp390 antibody inhibited *M. hyopneumoniae* from destroying and penetrating PTEC cells. The key functional domains of Mhp390 that interacts with host plasminogen were identified to be located within the residue region spanning from 425 to 604aa. These findings will deepen our understanding of the mechanisms underlying respiratory barrier invasion by *M. hyopneumoniae*, thereby facilitating the identification and development of therapeutic targets for *M. hyopneumoniae* infection. However, further investigation of respiratory barrier invasion by the Mhp390-mutant strain via in vivo studies is warranted.

## Supplementary Information

**Additional file 1. Multiple alignment of Mhp390 amino acid sequences among *Mycoplasma*** **species**. The sequences alignment was conducted by using the MEGA software and EMBL website. The resulting sequences alignment was visualized through Jalview. Highly conserved amino acid residues were highlighted with a dark blue background, and the depth of the color gradient was shown according to the percentage of identity. The length of the amino acid sequences was shown in parentheses.

**Additional file 2. Phylogenetic tree of the distribution and genetic distance of Mhp390 protein among *Mycoplasma* species**. This consensus tree of 1000 bootstrap replications was constructed based on Mhp390 amino acid sequences using the Maximum Likelihood method implemented in MEGA software (version 12.0). Branch colors were assigned according to the bootstrap values

**Additional file 3. Immunogenicity analysis of Mhp390 via iELISA**. The presence of anti-Mhp390 antibodies in serum from pigs naturally infected with *M. hyopneumoniae* or immunized with inactivated vaccine were identified via iELISA. The serum from healthy pig was served as the negative control.

**Additional file 4. The characterization of anti-Mhp390 monoclonal antibody.** (A) The specificity of anti-Mhp390 monoclonal antibody. Proteins of several different pathogens were tested through ELISA assay. (B) Indirect immunofluorescence assay analysis of anti-Mhp390 monoclonal antibody which recognized *M. hyopneumoniae* in the infected alveolar macrophages cells. Alveolar macrophages were treated with *M. hyopneumoniae *or and PBS, respectively. The nuclei‌ of PAMs were stained with DAPI (blue), while *M. hyopneumoniae* cells were labeled red by using anti-Mhp390 mAb and goat anti-mouse IgG-Cy3. *M. hyopneumoniae* cells were adhered to the edges of alveolar macrophages (white arrows). Scale bars =10 µm.

**Additional file 5. Strategies for artificial truncation of Mhp390**. (A) The secondary structure predicted through Phyre2. (B) Strategies for artificial truncation of rMhp390 protein. Based on the indicated truncation sites, the Mhp390 protein was artificially divided into three segments.

## Data Availability

The datasets used and analyzed during the current study are available from the corresponding author upon reasonable request.

## References

[1] Deffner P, Maurer R, Cvjetkovic V, Sipos W, Krejci R, Ritzmann M, Eddicks M (2022) Cross-sectional study on the in-herd prevalence of *Mycoplasma hyopneumoniae* at different stages of pig production. Vet Rec 191:e1317 35032397 10.1002/vetr.1317

[2] Fablet C, Marois-Crehan C, Simon G, Grasland B, Jestin A, Kobisch M, Madec F, Rose N (2012) Infectious agents associated with respiratory diseases in 125 farrow-to-finish pig herds: a cross-sectional study. Vet Microbiol 157:152–163 22226820 10.1016/j.vetmic.2011.12.015

[3] Assavacheep P, Thanawongnuwech R (2022) Porcine respiratory disease complex: dynamics of polymicrobial infections and management strategies after the introduction of the African swine fever. Front Vet Sci 9:1048861 36504860 10.3389/fvets.2022.1048861 PMC9732666

[4] Pageaut H, Lacouture S, Lehoux M, Marois-Crehan C, Segura M, Gottschalk M (2023) Interactions of *Mycoplasma hyopneumoniae* and/or *Mycoplasma hyorhinis* with *Streptococcus suis* serotype 2 using in vitro co-infection models with swine cells. Pathogens 12:866 37513713 10.3390/pathogens12070866 PMC10383509

[5] Galdeano JVB, Baraldi TG, Ferraz MES, de Souza Almeida HM, Mechler-Dreibi ML, Costa WMT, Montassier HJ, Mathias LA, de Oliveira LG (2019) Cross-sectional study of seropositivity, lung lesions and associated risk factors of the main pathogens of porcine respiratory diseases complex (PRDC) in Goias Brazil. Porcine Health Manag 5:23 31636919 10.1186/s40813-019-0130-0 PMC6791015

[6] Park C, Kang I, Seo HW, Jeong J, Choi K, Chae C (2016) Comparison of 2 commercial single-dose *Mycoplasma hyopneumoniae* vaccines and porcine reproductive and respiratory syndrome virus (PRRSV) vaccines on pigs dually infected with *M hyopneumoniae* and PRRSV. Can J Vet Res 80:112–123 27127338 PMC4836037

[7] Saade G, Deblanc C, Bougon J, Marois-Crehan C, Fablet C, Auray G, Belloc C, Leblanc-Maridor M, Gagnon CA, Zhu J, Gottschalk M, Summerfield A, Simon G, Bertho N, Meurens F (2020) Coinfections and their molecular consequences in the porcine respiratory tract. Vet Res 51:80 32546263 10.1186/s13567-020-00807-8 PMC7296899

[8] Cavagnero KJ, Gallo RL (2022) Essential immune functions of fibroblasts in innate host defense. Front Immunol 13:1058862 36591258 10.3389/fimmu.2022.1058862 PMC9797514

[9] Izadifar Z, Sontheimer-Phelps A, Lubamba BA, Bai H, Fadel C, Stejskalova A, Ozkan A, Dasgupta Q, Bein A, Junaid A, Gulati A, Mahajan G, Kim S, LoGrande NT, Naziripour A, Ingber DE (2022) Modeling mucus physiology and pathophysiology in human organs-on-chips. Adv Drug Deliv Rev 191:114542 36179916 10.1016/j.addr.2022.114542

[10] Sutherland TE, Dyer DP, Allen JE (2023) The extracellular matrix and the immune system: a mutually dependent relationship. Science 379:eabp8964 36795835 10.1126/science.abp8964

[11] Medcalf RL, Keragala CB (2021) The fibrinolytic system: mysteries and opportunities. Hemasphere 5:e570 34095754 10.1097/HS9.0000000000000570 PMC8171360

[12] Woolley LK, Fell SA, Djordjevic SP, Eamens GJ, Jenkins C (2013) Plasmin activity in the porcine airways is enhanced during experimental infection with *Mycoplasma hyopneumoniae*, is positively correlated with proinflammatory cytokine levels and is ameliorated by vaccination. Vet Microbiol 164:60–66 23490555 10.1016/j.vetmic.2013.02.003

[13] Wang J, Li Y, Pan L, Li J, Yu Y, Liu B, Zubair M, Wei Y, Pillay B, Olaniran AO, Chiliza TE, Shao G, Feng Z, Xiong Q (2021) Glyceraldehyde-3-phosphate dehydrogenase (GAPDH) moonlights as an adhesin in *Mycoplasma hyorhinis* adhesion to epithelial cells as well as a plasminogen receptor mediating extracellular matrix degradation. Vet Res 52:80 34082810 10.1186/s13567-021-00952-8 PMC8173509

[14] Wang J, Yu Y, Li Y, Li S, Wang L, Wei Y, Wu Y, Pillay B, Olaniran AO, Chiliza TE, Shao G, Feng Z, Xiong Q (2022) A multifunctional enolase mediates cytoadhesion and interaction with host plasminogen and fibronectin in *Mycoplasma hyorhinis*. Vet Res 53:26 35337383 10.1186/s13567-022-01041-0 PMC8951703

[15] Seymour LM, Jenkins C, Deutscher AT, Raymond BB, Padula MP, Tacchi JL, Bogema DR, Eamens GJ, Woolley LK, Dixon NE, Walker MJ, Djordjevic SP (2012) Mhp182 (P102) binds fibronectin and contributes to the recruitment of plasmin(ogen) to the *Mycoplasma hyopneumoniae* cell surface. Cell Microbiol 14:81–94 21951786 10.1111/j.1462-5822.2011.01702.x

[16] Seymour LM, Falconer L, Deutscher AT, Minion FC, Padula MP, Dixon NE, Djordjevic SP, Walker MJ (2011) Mhp107 is a member of the multifunctional adhesin family of *Mycoplasma hyopneumoniae*. J Biol Chem 286:10097–10104 21245147 10.1074/jbc.M110.208140 PMC3060461

[17] Jarocki VM, Santos J, Tacchi JL, Raymond BB, Deutscher AT, Jenkins C, Padula MP, Djordjevic SP (2015) MHJ_0461 is a multifunctional leucine aminopeptidase on the surface of *Mycoplasma hyopneumoniae*. Open Biol 5:140175 25589579 10.1098/rsob.140175 PMC4313372

[18] Xiong Q, Wang J, Ji Y, Ni B, Zhang B, Ma Q, Wei Y, Xiao S, Feng Z, Liu M, Shao G (2016) The functions of the variable lipoprotein family of *Mycoplasma hyorhinis* in adherence to host cells. Vet Microbiol 186:82–89 27016761 10.1016/j.vetmic.2016.01.017

[19] The HDOCK tool. https://hdock.phys.hust.edu.cn. Accessed 27 Mar 2024

[20] The HADDOCK tool. https://rascar.science.uu.nl/haddock2.4/. Accessed 29 Mar 2024.

[21] The Phyre2 tool. https://www.sbg.bio.ic.ac.uk/phyre2/html/page.cgi?id=index. Accessed 12 Aug 2023.

[22] The EMBL tool. https://www.ebi.ac.uk/jdispatcher/msa/tcoffee?stype=protein. Accessed 17 Apr 2025.

[23] The tvBOTtool. https://www.chiplot.online/tvbot.html. Accessed 9 Apr 2025.

[24] Xie J, Chen Y, Cai G, Cai R, Hu Z, Wang H (2023) Tree Visualization By One Table (tvBOT): a web application for visualizing, modifying and annotating phylogenetic trees. Nucleic Acids Res 51:W587–W592 37144476 10.1093/nar/gkad359 PMC10320113

[25] The iTOL tool. https://itol.embl.de/. Accessed 8 Apr 2025

[26] Liu W, Zhou D, Yuan F, Liu Z, Duan Z, Yang K, Guo R, Li M, Li S, Fang L, Xiao S, Tian Y (2019) Surface proteins mhp390 (P68) contributes to cilium adherence and mediates inflammation and apoptosis in *Mycoplasma hyopneumoniae*. Microb Pathog 126:92–100 30385395 10.1016/j.micpath.2018.10.035

[27] Minion FC, Adams C, Hsu T (2000) R1 region of P97 mediates adherence of *Mycoplasma hyopneumoniae* to swine cilia. Infect Immun 68:3056–3060 10769015 10.1128/iai.68.5.3056-3060.2000 PMC97530

[28] Liu W, Jiang P, Yang K, Song Q, Yuan F, Liu Z, Gao T, Zhou D, Guo R, Li C, Sun P, Tian Y (2022) *Mycoplasma hyopneumoniae* infection activates the NOD1 signaling pathway to modulate inflammation. Front Cell Infect Microbiol 12:927840 35873172 10.3389/fcimb.2022.927840 PMC9304885

[29] Liu W, Fang L, Li M, Li S, Guo S, Luo R, Feng Z, Li B, Zhou Z, Shao G, Chen H, Xiao S (2012) Comparative genomics of Mycoplasma: analysis of conserved essential genes and diversity of the pan-genome. PLoS One 7:e35698 22536428 10.1371/journal.pone.0035698 PMC3335003

[30] Kuhner S, van Noort V, Betts MJ, Leo-Macias A, Batisse C, Rode M, Yamada T, Maier T, Bader S, Beltran-Alvarez P, Castano-Diez D, Chen WH, Devos D, Guell M, Norambuena T, Racke I, Rybin V, Schmidt A, Yus E, Aebersold R, Herrmann R, Bottcher B, Frangakis AS, Russell RB, Serrano L, Bork P, Gavin AC (2009) Proteome organization in a genome-reduced bacterium. Science 326:1235–1240 19965468 10.1126/science.1176343

[31] Li S, Fang L, Liu W, Song T, Zhao F, Zhang R, Wang D, Xiao S (2019) Quantitative proteomic analyses of a pathogenic strain and its highly passaged attenuated strain of *Mycoplasma hyopneumoniae*. Biomed Res Int 2019:4165735 31355261 10.1155/2019/4165735 PMC6634062

[32] Lin H, Xu L, Yu S, Hong W, Huang M, Xu P (2020) Therapeutics targeting the fibrinolytic system. Exp Mol Med 52:367–379 32152451 10.1038/s12276-020-0397-x PMC7156416

[33] Raymond BB, Djordjevic S (2015) Exploitation of plasmin(ogen) by bacterial pathogens of veterinary significance. Vet Microbiol 178:1–13 25937317 10.1016/j.vetmic.2015.04.008

[34] Sebbane F, Uversky VN, Anisimov AP (2020) *Yersinia pestis* plasminogen activator. Biomolecules 10:1554 33202679 10.3390/biom10111554 PMC7696990

[35] Bhattacharya S, Ploplis VA, Castellino FJ (2012) Bacterial plasminogen receptors utilize host plasminogen system for effective invasion and dissemination. J Biomed Biotechnol 2012:482096 23118509 10.1155/2012/482096 PMC3477821

[36] Knaust A, Weber MV, Hammerschmidt S, Bergmann S, Frosch M, Kurzai O (2007) Cytosolic proteins contribute to surface plasminogen recruitment of *Neisseria meningitidis*. J Bacteriol 189:3246–3255 17307854 10.1128/JB.01966-06 PMC1855851

[37] Cork AJ, Jergic S, Hammerschmidt S, Kobe B, Pancholi V, Benesch JLP, Robinson CV, Dixon NE, Aquilina JA, Walker MJ (2009) Defining the structural basis of human plasminogen binding by streptococcal surface enolase. J Biol Chem 284:17129–17137 19363026 10.1074/jbc.M109.004317 PMC2719351

[38] Pieters M, Pijoan C, Fano E, Dee S (2009) An assessment of the duration of *Mycoplasma hyopneumoniae* infection in an experimentally infected population of pigs. Vet Microbiol 134:261–266 18835112 10.1016/j.vetmic.2008.08.016

[39] Jayaraman A, Walachowski S, Bosmann M (2024) The complement system: A key player in the host response to infections. Eur J Immunol 54:e2350814 39188171 10.1002/eji.202350814 PMC11623386

[40] Heggi MT, Nour El-Din HT, Morsy DI, Abdelaziz NI, Attia AS (2023) Microbial evasion of the complement system: a continuous and evolving story. Front Immunol 14:1281096 38239357 10.3389/fimmu.2023.1281096 PMC10794618

[41] Jia LJ, Gonzalez K, Orasch T, Schmidt F, Brakhage AA (2024) Manipulation of host phagocytosis by fungal pathogens and therapeutic opportunities. Nat Microbiol 9:2216–2231 39187614 10.1038/s41564-024-01780-0

[42] Boero E, Gorham RD Jr, Francis EA, Brand J, Teng LH, Doorduijn DJ, Ruyken M, Muts RM, Lehmann C, Verschoor A, van Kessel KPM, Heinrich V, Rooijakkers SHM (2023) Purified complement C3b triggers phagocytosis and activation of human neutrophils via complement receptor 1. Sci Rep 13:274 36609665 10.1038/s41598-022-27279-4 PMC9822988

[43] Sayyadi M, Hassani S, Shams M, Dorgalaleh A (2023) Status of major hemostatic components in the setting of COVID-19: the effect on endothelium, platelets, coagulation factors, fibrinolytic system, and complement. Ann Hematol 102:1307–1322 37074380 10.1007/s00277-023-05234-1 PMC10115391

[44] Heinen S, Hartmann A, Lauer N, Wiehl U, Dahse HM, Schirmer S, Gropp K, Enghardt T, Wallich R, Halbich S, Mihlan M, Schlotzer-Schrehardt U, Zipfel PF, Skerka C (2009) Factor H-related protein 1 (CFHR-1) inhibits complement C5 convertase activity and terminal complex formation. Blood 114:2439–2447 19528535 10.1182/blood-2009-02-205641

[45] Barthel D, Schindler S, Zipfel PF (2012) Plasminogen is a complement inhibitor. J Biol Chem 287:18831–18842 22451663 10.1074/jbc.M111.323287 PMC3365705

[46] Vieira ML, de Morais ZM, Vasconcellos SA, Romero EC, Nascimento AL (2011) In vitro evidence for immune evasion activity by human plasmin associated to pathogenic *Leptospira interrogans*. Microb Pathog 51:360–365 21802507 10.1016/j.micpath.2011.06.008

[47] Liu W, Jiang P, Song T, Yang K, Yuan F, Gao T, Liu Z, Li C, Guo R, Xiao S, Tian Y, Zhou D (2023) A recombinant chimera vaccine composed of LTB and *Mycoplasma hyopneumoniae* antigens P97R1, mhp390 and P46 elicits cellular immunologic response in mice. Vaccines 11:1291 37631860 10.3390/vaccines11081291 PMC10457768

[48] Law RH, Abu-Ssaydeh D, Whisstock JC (2013) New insights into the structure and function of the plasminogen/plasmin system. Curr Opin Struct Biol 23:836–841 24252474 10.1016/j.sbi.2013.10.006

