# Supplementary figures and images for: Mesomycoplasma hyopneumoniae lipoprotein Mhp390 serves as a plasminogen receptor mediating extracellular matrix degradation and respiratory epithelial cells injury
Source: Vet Res. 2025 Jun 21;56:124. doi: 10.1186/s13567-025-01551-7 (PMC12182672; doi:10.1186/s13567-025-01551-7)

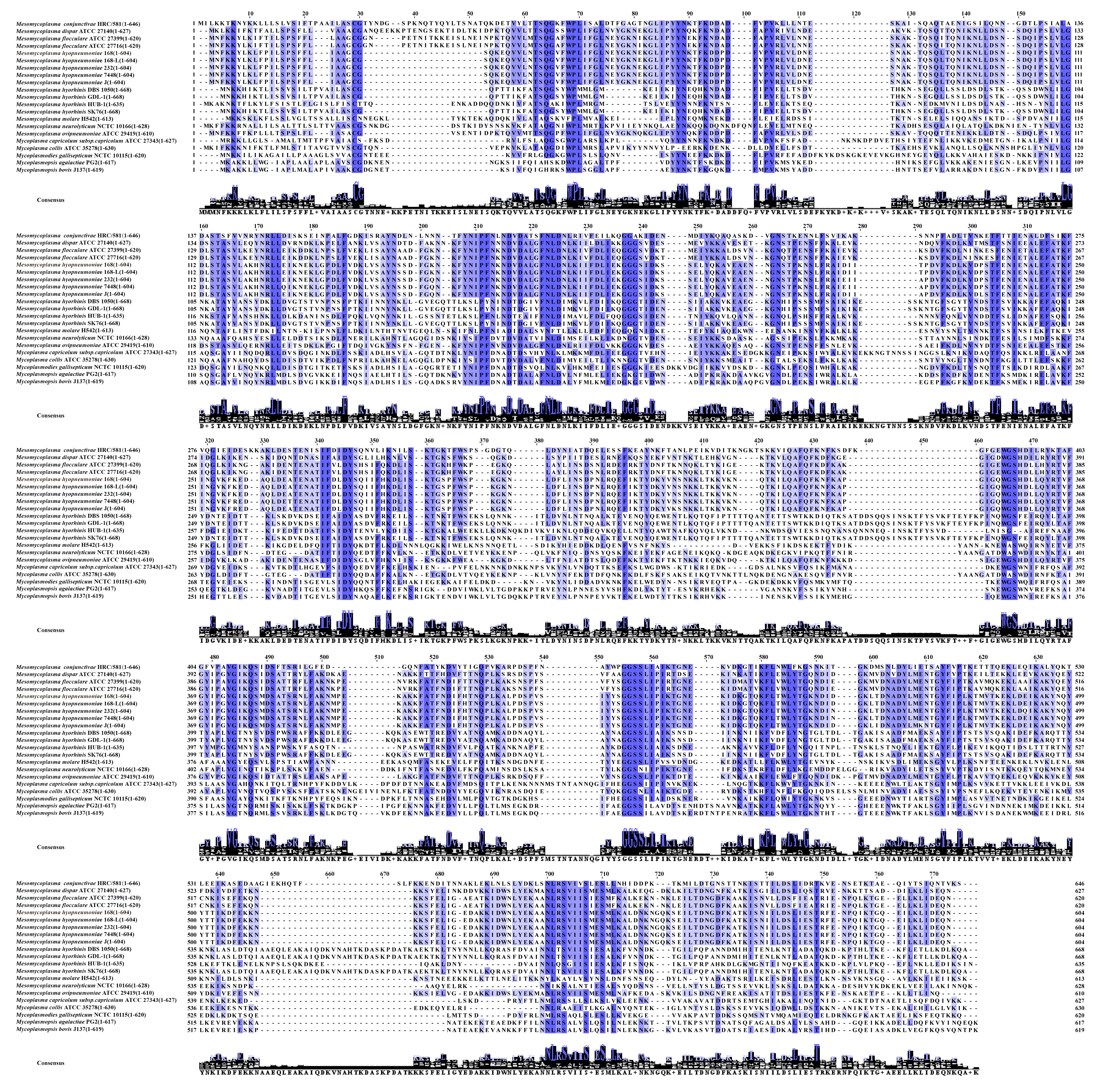

Supplement: Supplementary file 1 — Additional file 1. Multiple alignment of Mhp390 amino acid sequences among Mycoplasma species. The sequences alignment was conducted by using the MEGA software and EMBL website. The resulting sequences alignment was visualized through Jalview. Highly conserved amino acid residues were highlighted with a dark blue background, and the depth of the color gradient was shown according to the percentage of identity. The length of the amino acid sequences was shown in parentheses. [file 13567_2025_1551_MOESM1_ESM.tif]

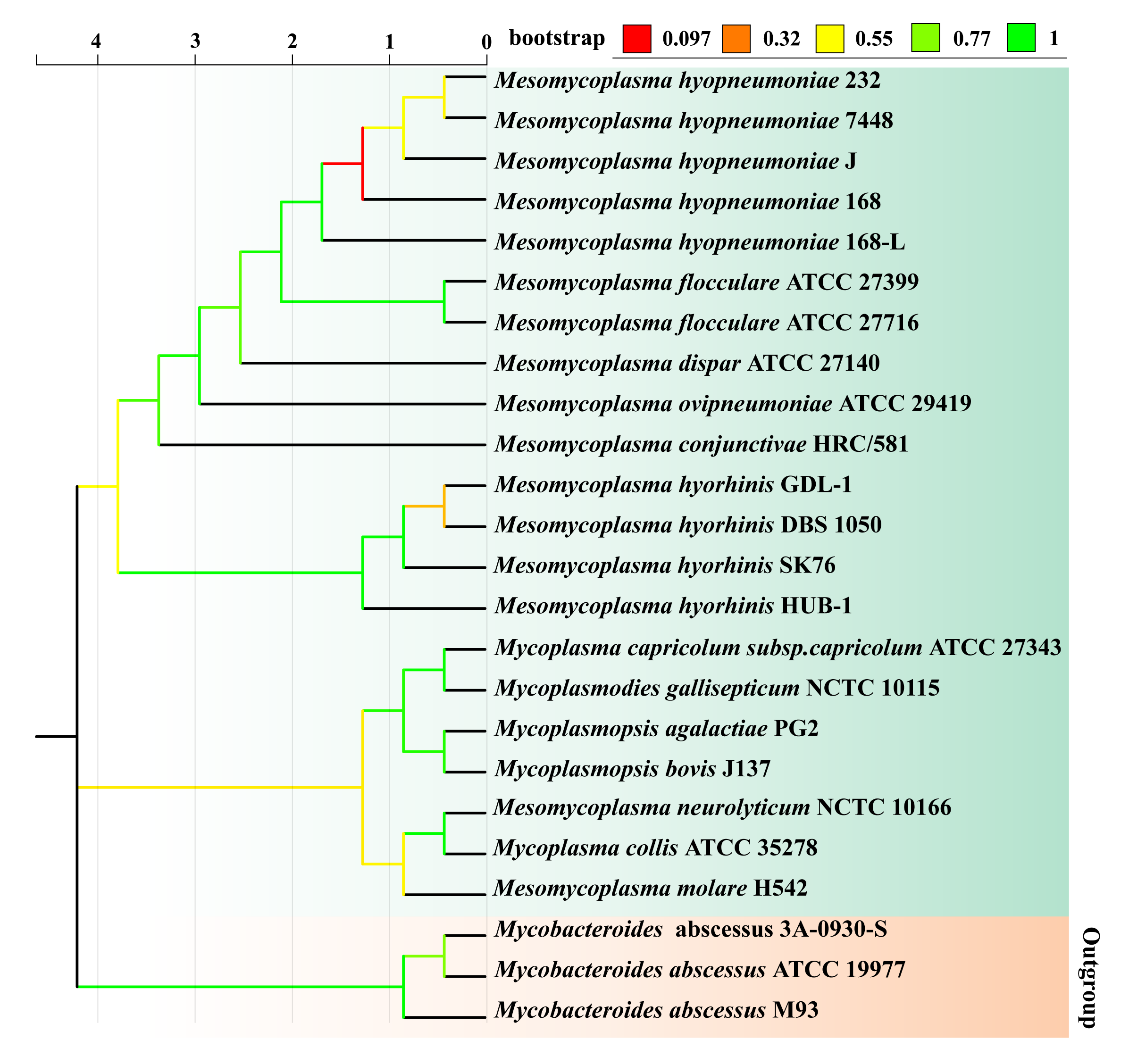

Supplement: Supplementary file 2 — Additional file 2. Phylogenetic tree of the distribution and genetic distance of Mhp390 protein among Mycoplasma species. This consensus tree of 1000 bootstrap replications was constructed based on Mhp390 amino acid sequences using the Maximum Likelihood method implemented in MEGA software (version 12.0). Branch colors were assigned according to the bootstrap values [file 13567_2025_1551_MOESM2_ESM.tif]

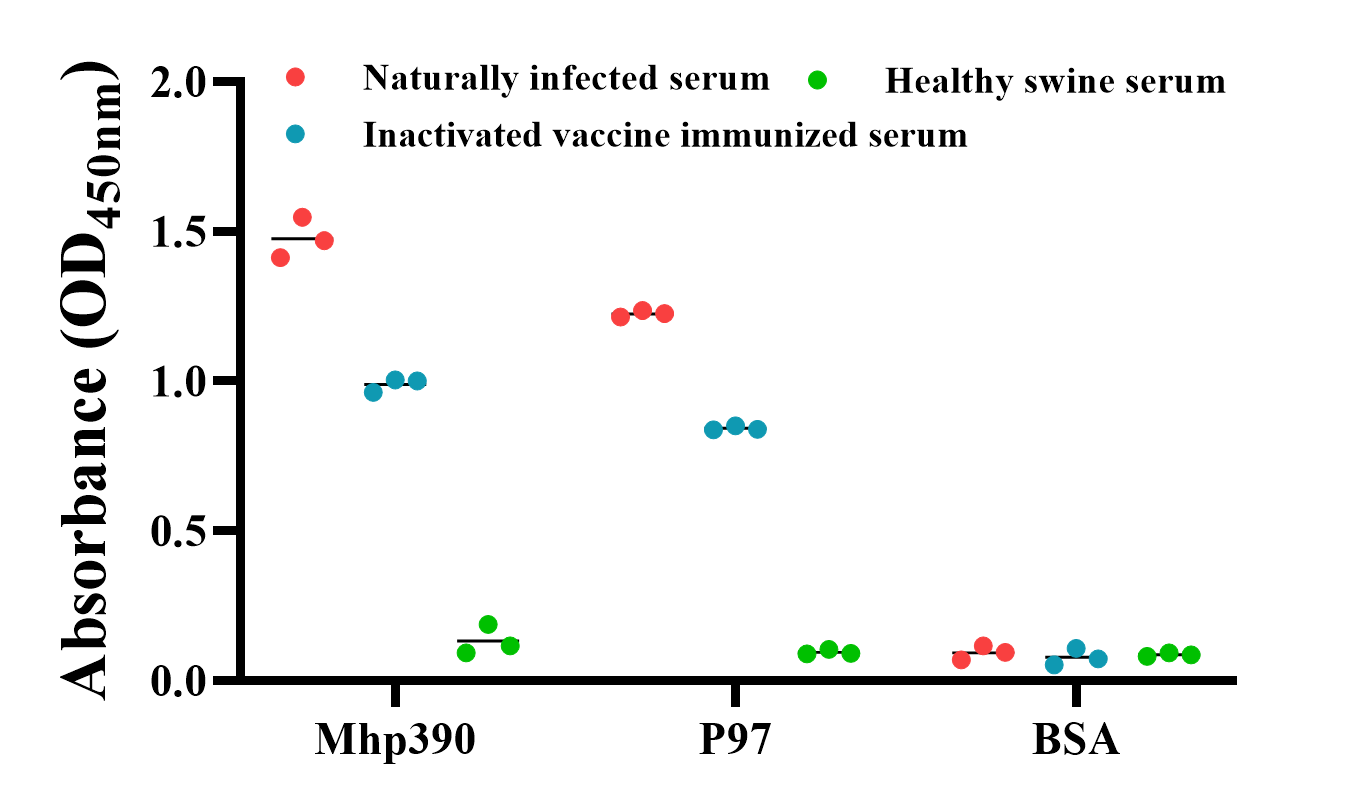

Supplement: Supplementary file 3 — Additional file 3. Immunogenicity analysis of Mhp390 via iELISA. The presence of anti-Mhp390 antibodies in serum from pigs naturally infected with M. hyopneumoniae or immunized with inactivated vaccine were identified via iELISA. The serum from healthy pig was served as the negative control. [file 13567_2025_1551_MOESM3_ESM.tif]

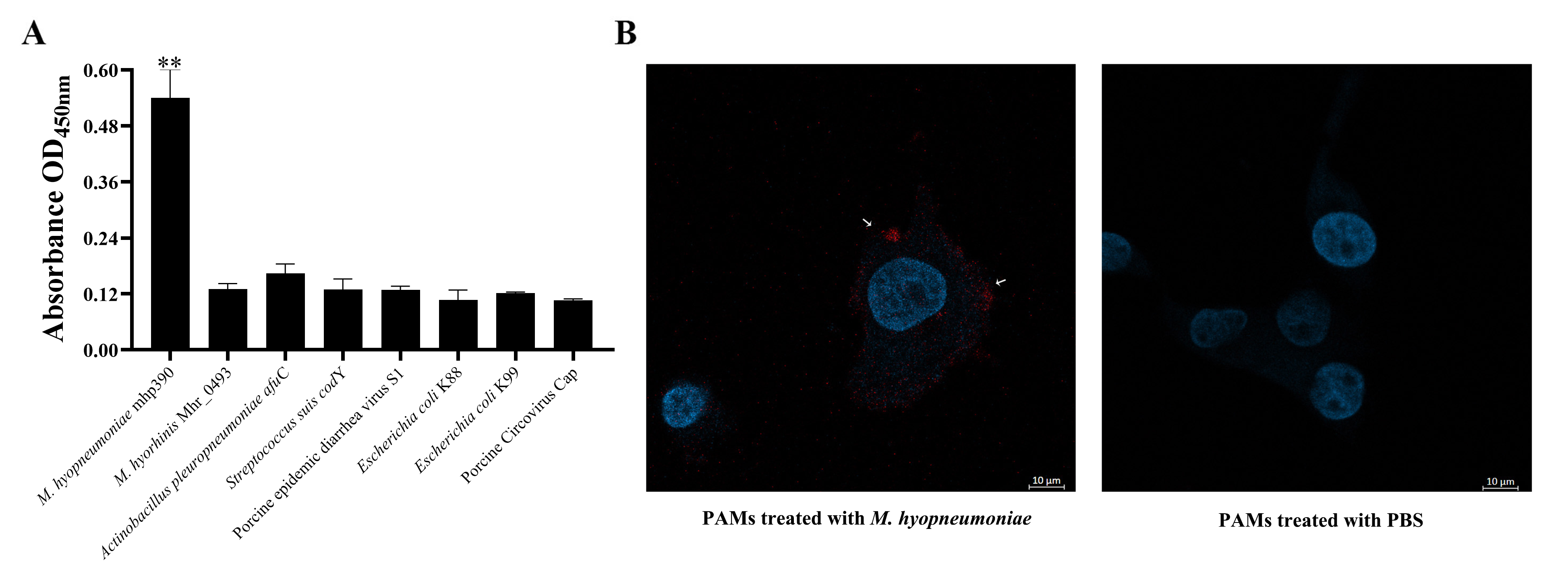

Supplement: Supplementary file 4 — Additional file 4. The characterization of anti-Mhp390 monoclonal antibody. (A) The specificity of anti-Mhp390 monoclonal antibody. Proteins of several different pathogens were tested through ELISA assay. (B) Indirect immunofluorescence assay analysis of anti-Mhp390 monoclonal antibody which recognized M. hyopneumoniae in the infected alveolar macrophages cells. Alveolar macrophages were treated with M. hyopneumoniae or and PBS, respectively. The nuclei‌ of PAMs were stained with DAPI (blue), while M. hyopneumoniae cells were labeled red by using anti-Mhp390 mAb and goat anti-mouse IgG-Cy3. M. hyopneumoniae cells were adhered to the edges of alveolar macrophages (white arrows). Scale bars =10 µm. [file 13567_2025_1551_MOESM4_ESM.tif]

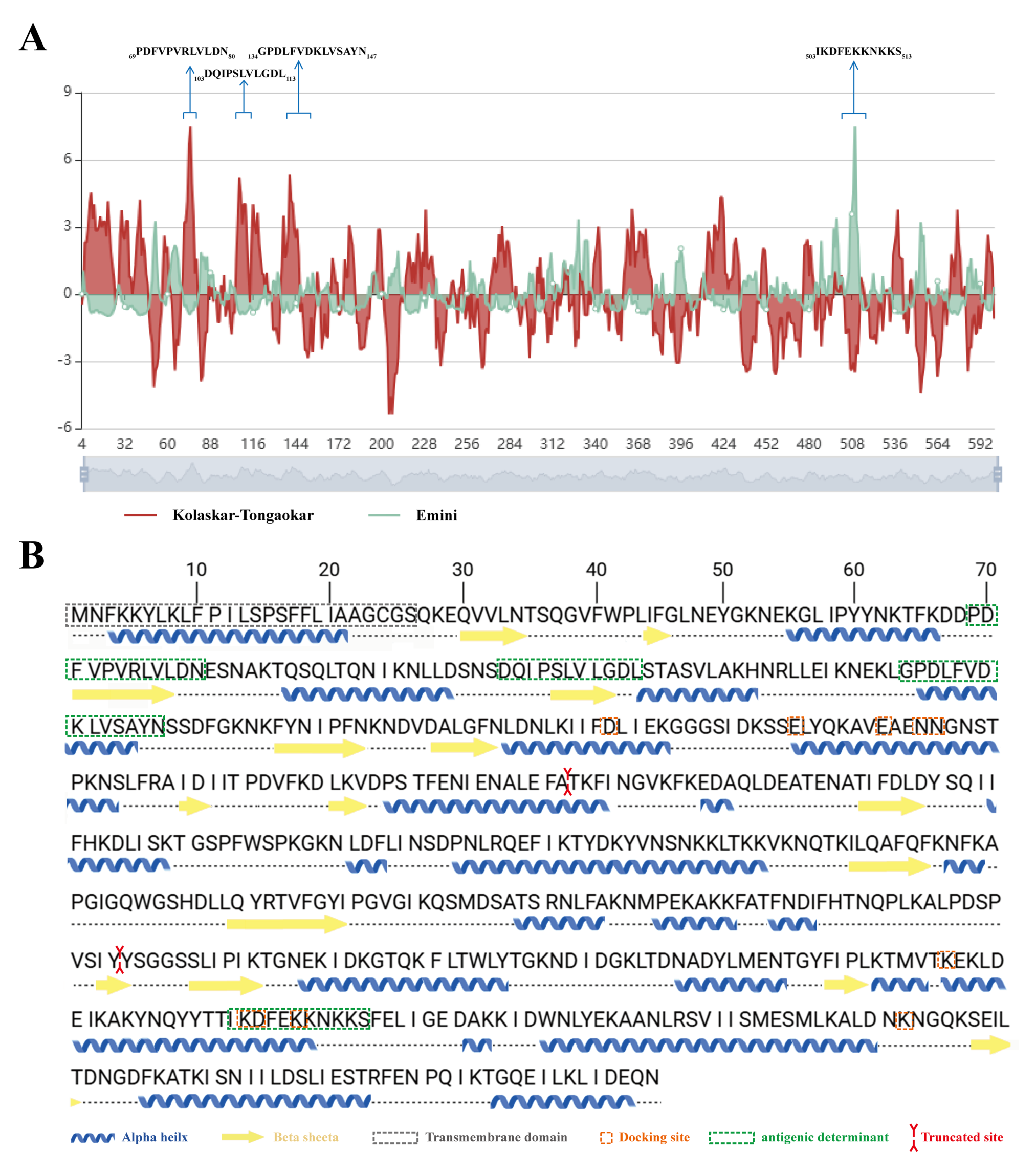

Supplement: Supplementary file 5 — Additional file 5. Strategies for artificial truncation of Mhp390. (A) The secondary structure predicted through Phyre2. (B) Strategies for artificial truncation of rMhp390 protein. Based on the indicated truncation sites, the Mhp390 protein was artificially divided into three segments. [file 13567_2025_1551_MOESM5_ESM.tif]
